# Anticipatory Burden in Adult-Child Caregivers: A Concept Analysis

**DOI:** 10.3390/healthcare10020356

**Published:** 2022-02-11

**Authors:** Hangying She, Yuncheng Man

**Affiliations:** 1 Frances Payne Bolton School of Nursing, Case Western Reserve University, Cleveland, OH 44106, USA; 2 Case School of Engineering, Case Western Reserve University, Cleveland, OH 44106, USA; yxm278@case.edu

**Keywords:** adult-child caregiver, anticipatory burden, caregiving burden

## Abstract

This study aims to analyze the concept of anticipatory burden in adult-child caregivers. A systematic literature review was performed using four databases, Pubmed, CINAHL, PsycINFO and Medline, with the keywords of “anticipatory burden” and “anticipated burden”. Simplified Wilson’s classic concept analysis modified by Walker and Avant was employed to identify the attributes, antecedents and consequences of anticipatory burden in the adult-child caregivers. Eighteen articles were analyzed. Attributes of anticipatory burden in adult-child caregivers were found to be: (1) subjective burden, (2) anticipation, (3) overestimation, (4) inability, and (5) family relationship. Antecedents were identified as: (1) potential care recipients, (2) caregiving willingness, and (3) a lack of resources. Consequences included: (1) prediction of caregiving willingness, (2) impacts on caregivers’ health, (3) intervention promotion, and (4) behavioral changes. As the adult-child caregiver is one of the main types of family caregivers for the fast-growing aging population, it is important to understand the attributes, antecedents, and consequences of their anticipatory burden. Based on the results of this study, resources such as intervention, policy, and counseling services are recommended to help adult-child caregivers lower their anticipatory burden and get better prepared for providing family care.

## 1. Introduction

Around 17% of all adult children end up providing care to their aging parents [1]. The adult-child caregivers can anticipate future caregiving burdens, which would negatively affect their own health and the care recipients. The concept of caregiving burden first appeared as family burden in the literature and was primarily associated with the healthcare cost borne by family members [2]. Nowadays, as the understanding of caregiver burden has evolved, it is described as a multifaceted phenomenon. Its definition now includes the concepts of objective burden (loss of time, energy, and social life), as well as subjective burden (emotional and relational stress) [3,4]. Moreover, financial burden is another important aspect of caregiving burden apart from the objective and subjective burden [5]. Caregiver burden has been extensively studied in the aforementioned three dimensions; however, for those adult children who are not yet caregivers but will become family caregivers for their aging parents in the future, the anticipatory burden of becoming future family caregivers is less understood.

Studying the anticipatory burden of adult-child caregivers is particularly crucial since the population aged over 65 is growing at a fast pace [6], and in many countries and cultures, adult children are obligated to provide care for their dependent parents [5,7,8,9]. For instance, Chinese parental respect is central in the Confucius culture. More importantly, as a retrospective report found that 37% to 39% of caregivers had considered the possibility of becoming caregivers before they actually carried out the role [10], adult children often plan to care for their aging parents before it happens, which significantly alters their current-state behaviors and even results in deteriorated physical and/or mental health conditions in their current stage [11].

The concept of anticipatory burden in caregiving has been developed in the literature, as mentioned in the concept analysis of anticipatory anxiety [11], anticipatory grief [12], anticipatory decision making [13], and pre-death grief [14]. However, none of these studies have provided a consistent conceptualization of anticipatory burden, specifically in the adult children population. Further, there is a lack of clarity between the existing definitions of what constitutes anticipatory burden in adult-child caregivers. Therefore, this study aims to provide a clear definition of the anticipatory burden in adult-child caregivers and a basis for its assessment and management.

## 2. Materials and Methods

### 2.1. Study Design

This systematic literature review uses a concept analysis that derives the attributes, antecedents, and consequences of anticipatory burden in adult-child caregivers, using simplified Wilson’s classic concept analysis [15] modified by Walker and Avant [16].

### 2.2. Literature Inclusion and Exclusion Criteria

This study was conducted in accordance with the guidelines of systematic literature reviews, following the Preferred Reporting Items for Systematic Review and Meta-Analysis (PRISMA) statement [17]. The inclusion criteria were English-language, peer-reviewed studies that appeared from literature search with contents or outcomes related to the keywords. The exclusion criteria were studies that lack appropriate subjects (e.g., the main subject is anticipatory/anticipated grief, anticipatory/anticipated anxiety, caregiver burden, or financial burden, etc.), contents or conceptual definitions, and degree theses were excluded.

### 2.3. Study Details

We searched “anticipatory burden” or “anticipated burden” in four databases—Pubmed, CINAHL, PsycINFO, and Medline—with keywords limited to the title and the abstract. Publications from 1 January 1982 to 1 December 2021 were searched, limited to those in the English language and peer-reviewed. Further, expert consultation and reference tracing were carried out for seeking potentially relevant publications. Moreover, regular alerts have been established for a few selected databases, such as PubMed, to update the literature review before manuscript submission.

**Figure 1:**
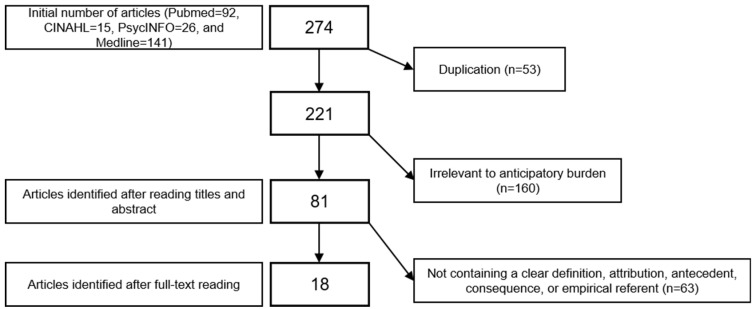
Flow diagram of study selection process.

The literature search was solely performed by a single author (H.S.) in this work. A total of 274 articles were extracted as primary sources using the keywords listed above. Following the exclusion of duplication, 221 secondary sources were extracted, and their titles and abstracts were carefully reviewed. Of these, 160 articles without mentioning anticipatory burden as a concept or without describing the phenomenon of anticipatory burden were excluded. Thereafter, following careful full-text screening, 63 articles without a clear definition, attribute, antecedent, consequence, or empirical referent were excluded. Finally, 18 articles were included in this study (Figure 1). The specific process is as follows:

- Select a concept.
  The concept of anticipatory burden in adult-child caregivers was selected.
- Determine the aims of the concept analysis.
  The aim of this study is to provide a clear definition of the anticipatory burden in adult-child caregivers and a basis for its assessment and management.
- Identify previous definitions and uses of the concept.
  Two previous definitions and five uses of the concept in different contexts were identified by reviewing seven of the included articles.
- Determine the defining attributes.
  Five defining attributes were determined. Subjective burden is based on the nature of anticipation, where the expected burden-generating events have not happened yet. Anticipation is based on the nature of this concept, as mentioned by Laditka, S.B. and M. Pappas-Rogich [11]. Overestimation is based on the inaccuracy of anticipation, as mentioned by Huang et al. [18]. Inability is based on the nature of burden that will be generated when adult-children are unable to tackle all the caregiving demands. The family relationship was mentioned by Feeney, J.A. and L. Hohaus [19], as a family relationship will influence the willingness (Antecedents) of caregiving.
- Present a model case of the concept.
- Present borderline, related, contrary, invented, and illegitimate cases.
- Identify antecedents and consequences of the concept.
  Three antecedents were identified: Potential care recipients, caregiving willingness, and a lack of resources. Four consequences were identified: Prediction of caregiving willingness, impacts on caregivers’ health, intervention promotion, and behavioral changes.
- Define empirical referents.

## 3. Results

### 3.1. Previous Definitions and Uses of Anticipatory Burden

Anticipatory burden has been defined and used elsewhere. Feeney et al. described anticipated burden as the burden of spouse caregivers who perceived their partners in severe health conditions in the future [19]. Sekhon et al. studied defined anticipatory burden in a random control trial (RCT) as the accessibility of the intervention and the required amount of effort for participation [20]. Notably, in this study, the concept of anticipatory burden was utilized to determine if low accessibility is a significant factor leading to reduced participation or non-adherence to the intervention among the participants. Another study by Naidoo et al. confirmed that reducing anticipatory burden in RCT could encourage participation [21]. Furthermore, Wilson et al. described the anticipatory burden in the context of anticipatory prescribing, where nurses still experience emotional burden even though the medication was administrated with considerable caution [22]. Additionally, Huang et al. described the impact of anticipatory financial burden on a patient’s decision to undergo contralateral prophylactic mastectomy [18]. The recent COVID-19 pandemic has brought up anticipatory burden in various aspects. Kashyap et al. described the anticipatory burden arising in the healthcare system during the COVID-19 pandemic [23]. Kozloff et al. used anticipatory burden to describe the burden of people with schizophrenia and related disorders due to the COVID-19 pandemic [24].

### 3.2. Attributes of Anticipatory Burden in Adult-Child Caregivers

**Figure 2:**
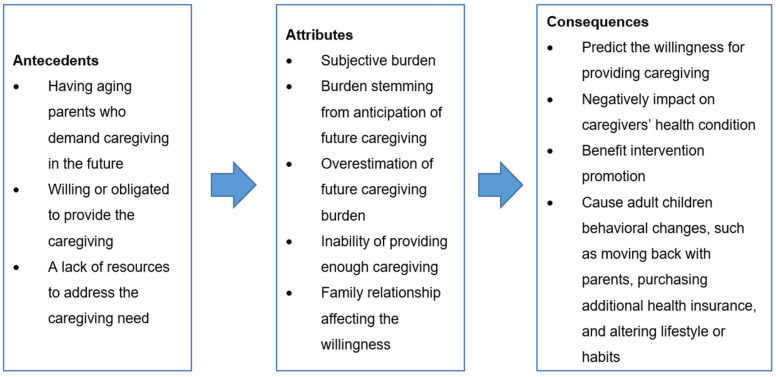
Concept diagram of “anticipatory burden in adult-child caregivers”.

According to the concept analysis procedural [16], determining the defining attributes of a concept is the core of concept analysis. Defining attributes are the essential characteristics of a concept. It helps differentiate the concept of anticipatory burden in adult-child caregivers from other related concepts. The following attributes of anticipatory burden in adult-child caregivers were identified by reviewing various pieces of literature (Figure 2).

(1) Subjective burden

Adult children who anticipate taking care of their aged parents in the future have not entered the family caregiver role; thus, their anticipatory burden is purely subjective and caused by emotional and relational stress. Since they do not undertake caregiving tasks at the current stage, no objective burden is associated with the anticipatory burden.

(2) Anticipation

The anticipatory burden in adult-child caregivers is mainly tied to the anticipation of the caregiving burden in the future, whether foreseeable or addressable at the current stage [11]. Adult children often start planning ahead and are stressed by the objective, subjective, and financial burdens of providing caregiving to their aging parents. It is necessary to provide practical social support to adult children undertaking the family caregiver role to help them identify what they need and better prepare them both physically and mentally.

(3) Overestimation

As described by Huang at al., there is a mismatch between the anticipatory burden and the actual burden when patients decide to have their surgeries [18]. Similarly, adult children sometimes overestimate the caregiving burden for their parents before they actually undertake the family caregiver role. This is primarily due to the fact that they lack certain knowledge on aging population healthcare and also resources.

(4) Inability

Once adult children enter the role of the family caregiver and provide caregiving to their parents, they have to carry out the duty on a constant basis. As a result, they may anticipate that they are unable to maintain such care on the long run and therefore experience anticipatory burden. On one hand, there is a lack of resourcefulness in the adult children; on the other hand, they may have overestimated the actual caregiving burden.

(5) Family relationship

A tight family relationship can increase the willingness of adult children to take care of their parents [19]. Anticipatory burden is generated only if the adult children decide to care their aging parents in the future, and thus, the family relationship is an important factor here.

### 3.3. Cases

#### 3.3.1. Model Case

A model case refers to a case that presents all the key attributes of the concept [16]. This study presents two model cases based on the observation of daily life.

A 45 year-old man lives with his wife and two school-age daughters. Both him and his wife are employed full-time. His father has lived alone for more than 20 years and has recently been diagnosed with early stage dementia. After being aware of the trajectory of dementia (the continuous deteriorating physical and mental health condition of his father) and the future increasing caregiving burden that accompanied it, he was stressed so much that he could not sleep for a week because of the anticipatory burden, such as the caring demands, life–work balance issues, and decision-making conflicts in the future.

On the other hand, a 30-year-old women is employed full-time and unmarried. She lives in town, while her parents live in a rural area. Her mother had cardiovascular disease for more than 5 years, and his father has lived with diabetes for more than 10 years. Both of her parents are still able to take care of themselves. However, she can foresee the deep waters she will be in as her parents are growing older and their health condition continues to drop as the diseases develop. Should she move back with her parents to take care of them and give up her career, or keep her job and leave her parents to the paid care assistant they may barely know? Moreover, leaving her parents to someone unknown leads to more emotional burden due to blame and guilt generated by the filial piety culture. The anticipatory burden and the conflict between the two decisions makes her worried and stressed.

#### 3.3.2. Contrary Case

A contrary case does not display attributes of the concept but instead presents attributes contrary to it. Its presentation allows the attributes of the concept being used to be better understood and clarified.

A 78-year-old woman lives in a nursing home. She feels isolated one day and wants the company of a nurse who used to take good care of the old woman. However, the nurse recently left this job and therefore did not respond to the demand. In this case, the old woman has emotional demand, but the nurse, the previous formal caregiver of her, does not have anticipatory burden, and therefore she simply ignored the demand.

#### 3.3.3. Related Case

A related case contains part of the key attributes [16]. For example, the anticipatory grief. Lindemanm, a psychiatrist, shows in his article [25] that a wife refused her husband returning home following retiring from the military. The reason is that the wife did not anticipate the husband surviving from the war and had gone through the anticipatory grief and psycho-emotionally abandoned the marital relationship with him.

#### 3.3.4. Borderline Case

A borderline case includes some of the attributes of the concept but not all [16]. In this study, the borderline case addresses the attributes of subjective burden, anticipation, and inability.

An employee recently lost his job, and he can no longer afford health insurance. He takes certain medication that was covered by health insurance, and he now has to pay from his own pocket. He is frustrated about not being able to find a new job to cover his bills; he anticipates that if he cannot find a job soon, he will soon go bankrupt.

#### 3.3.5. Invented Case

An invented case is demonstrated by the concept being taken out of the context of our own experience [16]. In the context of parenting, the parents experience anticipatory burden by anticipating issues in educational competition, physical and mental development, and campus bullying as their children grow up.

### 3.4. Identification of Antecedents and Consequences of Anticipatory Burden in Adult-Children Caregivers

#### 3.4.1. Antecedents

Antecedents refer to additional conditions or events before the occurrence of the concept. Based on the literature review, the following antecedents were identified:

(1) Potential care recipients;
(2) Caregiving willingness;
(3) A lack of resources.

First, the aging parents may have continuously deteriorating health conditions and therefore demand caregiving from their children. Second, adult children are willing or obligated to provide caregiving to their aging parents regardless of whether they have enough resources. Third, adult children experience anticipatory burden in caring for their aging parents due to a lack of resources for addressing their needs.

#### 3.4.2. Consequences

Consequences are those elements or conditions that occur as a result of the concept [16]. Based on the literature review, the following consequences were identified:

(1) Prediction of caregiving willingness;
(2) Impacts on caregivers’ health;
(3) Intervention promotion;
(4) Behavioral changes.

Feeney et al. found that anticipatory burden is associated with the willingness to take care of a spouse or partner [19]. Similarly, the anticipatory burden provides a reliable prediction of the willingness of adult children to care for their aging parents. Moreover, anticipatory burden can negatively impact adult-child caregivers’ health, as indicated by the research of Kumari et al., as subjective burden often leads to poor physical and mental health [26]. Further, based on evidence by Rizzieri et al. showing that the availability of less-burdensome therapies reduced the following caregiving burden, the reduction in the anticipatory burden can benefit intervention promotion in both the adult-child caregivers and the aging parents [27]. Last but not least, adult children change their behaviors after they experience the anticipatory burden of taking care of their aging parents. For example, they may correct their parents’ bad habits or unhealthy lifestyle [28], or they may purchase additional medical insurance for their parents due to the financial pressure of medical care [29].

### 3.5. Definition of Anticipatory Burden in Adult-Children Caregivers

**Figure 3:**
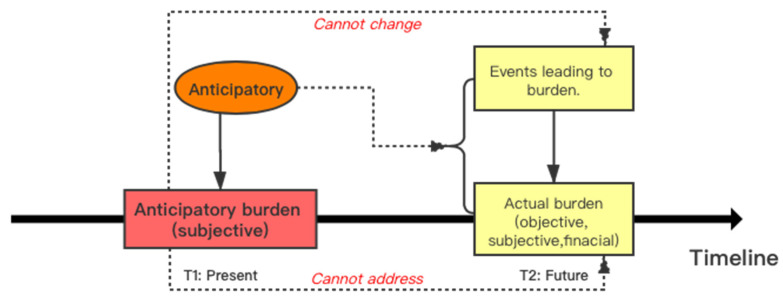
Theoretical model of anticipatory burden in adult-child caregivers.

Based on the previous definitions of anticipatory burden and merging them with the key attributes identified in this study, the anticipatory burden (subjective) was generated by anticipating two things in the future (Figure 3). Firstly, the adult-children anticipate that they cannot change the events/facts of aging and health deterioration of their parents. Both aging and health deterioration of their parents lead to potential caregiver burden in the future. Secondly, the adult-children anticipate that they cannot address the caregiver burden (objective) in the future generated by the aforementioned events/facts.

### 3.6. Empirical Referents

Empirical referents are used in the final step in the conceptual analysis to recognize the characteristics of the defining attributes and to show that the properties of the concept exist in the actual field [16]. They are particularly useful to address the question: “If we are going to measure this concept or determine its existence in the real world, how do we do so?” Existing instruments for measuring caregiving burden are mostly based on existing objectives [30,31,32,33,34]; they are not designed to determine subjective burden, such as the anticipatory burden in adult-child caregivers. The Zarit Burden Interview is the most popular instrument for determining caregiver burden, especially subjective burden [35]. This self-report instrument contains 22 items assessing the perceived burden in caregivers. However, it has not been applied to determine anticipatory burden in adult-child caregivers.

## 4. Discussion

This concept analysis concluded that the anticipatory burden in adult-child caregivers is defined as the subjective burden stemming from their planning to take care of their aging parents in the future. Three antecedents, five attributes, and four consequences of the anticipatory burden in adult-child caregivers were generated (Table 1).

Three antecedents of anticipatory burden were generated. Two of them were generated from previous definitions and uses: (1) Specific events will happen in future; (2) These events may not be addressed. In the context of adult-child caregiver, these two antecedents will be (1) Caregiving willingness; (2) Lacking resources. The third antecedent, “Potential care recipients”, was based on the specific context—the adult-child caregiver. This finding could help researchers design inclusion criteria when recruiting the target population for a study about anticipatory burden in adult-child caregivers. Additionally, researchers could also develop or promote interventions to help adult children release their anticipatory burden, such as distributing information about how to promote their parent(s)’ well-being and about how to obtain caregiving resources.

Five attributes of anticipatory burden were generated. The “subjective burden”, “anticipation”, and “inability” could help develop items in the measurement instrument. For example, “subjective burden” could be reflected in the language “Do you feel/think/believe…”; “anticipation” could be reflected in the language “the potential event(s) in the future”; “inability” could be reflected as “something is out of control/lack of resources to something/cannot handle something”. The “family relationship” and “overestimation” could be used in the covariates or factors analyses of anticipatory burden in the future study.

Four consequences of anticipatory burden were generated. Though anticipatory burden is based on the willingness to be an adult-child caregiver, an overly high anticipatory burden may decrease the willingness and make the adult children become hesitant when they consider taking on the caregiver role. It may also impact adult children’s health, such as anxiety, worry, and sleep problems, etc. These two negative consequences can be demonstrated as the problems needing to be tacked in policy-making. They can also be used as a reference in mental health consulting if the clients may have caregiving concerns. The anticipatory burden could also help promote interventions. There is no existing intervention target at the potential adult-child caregiver. However, there are lots of interventions about caregivers. The existing intervention could be promoted or revised into a new version to help the potential adult-child caregiver release their anticipatory burden. The last consequence, which is behavior changes, can also help as a reference to develop or promote the interventions.

A limitation of this work is that only a few terms were used when searching the database. Other terms such as “anticipatory grief” may help retrieve more studies for the conceptualization. Another limitation is that some key aspects such as cultural differences and differences in family structure, varying from country to country and family to family, were not analyzed. Future work will preferentially focus on measuring thoughts, feelings, daily experiences, and health of adult-child caregivers from diversified backgrounds to determine their caregiving demands, perceived stress, resourcefulness, and negative physical or physiological symptoms (if any).

## 5. Conclusions

As taking care of an increasingly aging population presents a global challenge, it is important to study and understand family caregivers who will have a central role in caring for the aging population. This study provides a conceptual analysis that identifies the meaning and properties of anticipatory burden in adult-child caregivers. The definition of anticipatory burden in adult-child caregivers was found as the present subjective burden that adult-children are perceiving, which stems from their plan to take care of their parents and the resulting burden in the future. Based on these findings, resources such as intervention, policy, and counseling services are recommended to help the adult-children to reduce their anticipatory burden and be better prepared to become family caregivers.

## Figures and Tables

**Table 1:** Previous definitions and uses, attributes, antecedents, attributes, and consequences of anticipatory burden in adult-child caregivers.

Dimension	Sub-Dimensions	Key Findings in the Literature
Previous Definitions and Uses	Definitions	(1) In the context of spousal caregivers; (2) In the context of random control trial participants. [19,20]
	Uses	(1) In the context of random control trial participants; (2) In the context of anticipatory prescribing by nurses; (3) In the context of anticipatory financial burden on a patient’s decision; (4) In the context of the healthcare system during the COVID-19 pandemic; (5) In the context of people with schizophrenia and related disorders due to the COVID-19 pandemic. [18,21,22,23,24]
Attributes	Subjective burden	N.A.
	Anticipation	The anticipatory burden in adult-child caregivers is mainly tied to the anticipation of the caregiving burden in the future. [11]
	Overestimation	The mismatch between the anticipatory burden and the actual burden. [18]
	Inability	N.A.
	Family relationship	A tight family relationship can increase the willingness of adult children for taking care of their parents. [19]
Antecedents	Potential care recipients	N.A.
	Caregiving willingness	N.A.
	A lack of resources	N.A.
Consequences	Prediction of caregiving willingness	The anticipatory burden is associated with the willingness of taking care of a spouse or partner. [19]
	Impacts on caregivers’ health	The anticipatory burden can negatively impact adult-child caregivers’ health. [26]
	Intervention promotion	The availability of less-burdensome therapies reduced the following caregiving burden. [27]
	Behavioral changes	(1) correct parents’ bad habits or unhealthy lifestyles; (2) purchase additional medical insurance for their parents. [28,29]
Empirical Referents		Existing instruments for measuring caregiving burden are mostly based on existing objectives. The Zarit Burden Interview determines subjective caregiver burden, however, has not been applied in the anticipatory burden in adult-child caregivers. [30,31,32,33,34,35]

## Data Availability

Not applicable.

## References

[1] Wettstein G., Zulkarnain A. (2017). How Much Long-Term Care Do Adult Children Provide.

[2] Grad J., Sainsbury P. (1966). Problems of caring for the mentally ill at home. Proc. R. Soc. Med..

[3] Walker A.J., Pratt C.C., Eddy L. (1995). Informal caregiving to aging family members: A critical review. Fam. Relat..

[4] Montgomery R.J., Gonyea J.G., Hooyman N.R. (1985). Caregiving and the experience of subjective and objective burden. Fam. Relat..

[5] Zhan H.J. (2002). Chinese caregiving burden and the future burden of elder care in life-course perspective. Int. J. Aging Hum. Dev..

[6] Weisman J. (2005). Aging Population Poses Global Challenges.

[7] Cicirelli V.G. (1993). Attachment and obligation as daughters’ motives for caregiving behavior and subsequent effect on subjective burden. Psychol. Aging.

[8] Lee Y.-R., Sung K.-T. (1998). Cultural influences on caregiving burden: Cases of Koreans and Americans. Int. J. Aging Hum. Dev..

[9] Togonu-Bickersteth F. (1989). Conflicts over caregiving: A discussion of filial obligations among adult Nigerian children. J. Cross-Cult. Gerontol..

[10] Chappell N., Litkenhaus R. (1995). Informal Caregivers to Adults in British Columbia.

[11] Laditka S.B., Pappas-Rogich M. (2001). Anticipatory caregiving anxiety among older women and men. J. Women Aging.

[12] Fulton R., Gottesman D.J. (1980). Anticipatory grief: A psychosocial concept reconsidered. Br. J. Psychiatry.

[13] Slyer J.T., Archibald E., Moyo F., Truglio-Londrigan M. (2018). Advance care planning and anticipatory decision making in patients with Alzheimer disease. Nurse Pract..

[14] Lindauer A., Harvath T.A. (2014). Pre-death grief in the context of dementia caregiving: A concept analysis. J. Adv. Nurs..

[15] Wilson J. (1963). Thinking with Concepts.

[16] Walker L.O., Avant K.C. (2005). Strategies for Theory Construction in Nursing.

[17] Page M.J., McKenzie J.E., Bossuyt P.M., Boutron I., Hoffmann T.C., Mulrow C.D., Shamseer L., Tetzlaff J.M., Akl E.A., Brennan S.E. (2021). The PRISMA 2020 statement: An updated guideline for reporting systematic reviews. BMJ.

[18] Huang J., Chagpar A. (2018). Impact of anticipated financial burden on patient decision to undergo contralateral prophylactic mastectomy. Surgery.

[19] Feeney J.A., Hohaus L. (2001). Attachment and spousal caregiving. Pers. Relatsh..

[20] Sekhon M., Cartwright M., Lawes-Wickwar S., McBain H., Ezra D., Newman S., Francis J.J. (2021). Does prospective acceptability of an intervention influence refusal to participate in a randomised controlled trial? An interview study. Contemp. Clin. Trials Commun..

[21] Naidoo N., Nguyen V.T., Ravaud P., Young B., Amiel P., Schante D., Clarke M., Boutron I. (2020). The research burden of randomized controlled trial participation: A systematic thematic synthesis of qualitative evidence. BMC Med..

[22] Wilson E., Morbey H., Brown J., Payne S., Seale C., Seymour J. (2015). Administering anticipatory medications in end-of-life care: A qualitative study of nursing practice in the community and in nursing homes. Palliat. Med..

[23] Kashyap S., Gombar S., Yadlowsky S., Callahan A., Fries J., Pinsky B.A., Shah N.H. (2020). Measure what matters: Counts of hospitalized patients are a better metric for health system capacity planning for a reopening. J. Am. Med. Inform. Assoc..

[24] Kozloff N., Mulsant B.H., Stergiopoulos V., Voineskos A.N. (2020). The COVID-19 Global Pandemic: Implications for People with Schizophrenia and Related Disorders. Schizophr. Bull..

[25] Lindemann E. (1944). Symptomatology and management of acute grief. Am. J. Psychiatry.

[26] Kumari R., Kohli A., Malhotra P., Grover S., Khadwal A. (2018). Burden of caregiving and its impact in the patients of acute lymphoblastic leukemia. Ind. Psychiatry J..

[27] Rizzieri A.G., Verheijde J.L., Rady M.Y., McGregor J.L. (2008). Ethical challenges with the left ventricular assist device as a destination therapy. Philos. Ethics Humanit. Med..

[28] Oliveira D., Sousa L., Orrell M. (2019). Improving health-promoting self-care in family carers of people with dementia: A review of interventions. Clin. Interv. Aging.

[29] Chandra A., Young J.S., Dalle Ore C., Dayani F., Lau D., Wadhwa H., Rick J.W., Nguyen A.T., McDermott M.W., Berger M.S. (2019). Insurance type impacts the economic burden and survival of patients with newly diagnosed glioblastoma. J. Neurosurg..

[30] Appleton S., Adams R., Porter S., Peacock M., Ruffin R. (2003). Sustained improvements in dyspnea and pulmonary function 3 to 5 years after lung volume reduction surgery. Chest.

[31] Cedano S., Bettencourt A.R., Traldi F., Machado M.C., Belasco A.G. (2013). Quality of life and burden in carers for persons with chronic obstructive pulmonary disease receiving oxygen therapy. Rev. Lat.-Am. Enferm..

[32] Elmståhl S., Malmberg B., Annerstedt L. (1996). Caregiver’s burden of patients 3 years after stroke assessed by a novel caregiver burden scale. Arch. Phys. Med. Rehabil..

[33] Lee E., Lum C.M., Xiang Y.T., Ungvari G.S., Tang W.K. (2010). Psychosocial condition of family caregivers of patients with chronic obstructive pulmonary disease in Hong Kong. East Asian Arch. Psychiatry.

[34] Pinto R.A., Holanda M.A., Medeiros M.M., Mota R.M., Pereira E.D. (2007). Assessment of the burden of caregiving for patients with chronic obstructive pulmonary disease. Respir. Med..

[35] Zarit S.H., Reever K.E., Bach-Peterson J. (1980). Relatives of the impaired elderly: Correlates of feelings of burden. Gerontologist.

